# Brainard District Medical Society

**Published:** 1878-01

**Authors:** 


The BRAINARD DISTRICT MEDICAL SOCIETY, met in November, 1877, with thirteen members in attendance. Dr. M. Hurst, of Sweetwater, read a paper describing a case of compound fracture of the tibia and fibula; Dr. H. B. Brown reported a case of popliteal aneurism; Dr. J. D. Whitley, of Oakford, reported a case of belladonna poisoning; Dr. J. P. Walker reported a case of tubal pregnancy; Dr. M. Hurst described two cases of shoulder presentation; Dr. Walker read a communication from Dr. Jennings, of Delaware, containing a report of a case of vesico-vaginal fistula, and a paper by himself on the use of the obstetric forceps.

A resolution was adopted, making the CHICAGO MEDICAL JOURNAL AND EXAMINER the official organ of the association.

